# How Safe Is Ginger Rhizome for Decreasing Nausea and Vomiting in Women during Early Pregnancy?

**DOI:** 10.3390/foods7040050

**Published:** 2018-04-01

**Authors:** Julien Stanisiere, Pierre-Yves Mousset, Sophie Lafay

**Affiliations:** GYNOV SAS, 5 rue Salneuve, 75017 Paris, France; j.stanisiere@gynov.com (J.S.); py.mousset@jenwin.fr (P.-Y.M.)

**Keywords:** pregnancy, *Zingiber officinale* R, ginger, NVP, toxicity, safety, adverse effects, food supplement, CAM

## Abstract

Ginger, *Zingiber officinale* Roscoe, is increasingly consumed as a food or in food supplements. It is also recognized as a popular nonpharmacological treatment for nausea and vomiting of pregnancy (NVP). However, its consumption is not recommended by all countries for pregnant women. Study results are heterogeneous and conclusions are not persuasive enough to permit heath care professionals to recommend ginger safely. Some drugs are also contraindicated, leaving pregnant women with NVP with few solutions. We conducted a review to assess effectiveness and safety of ginger consumption during early pregnancy. Systematic literature searches were conducted on Medline (via Pubmed) until the end of December 2017. For the evaluation of efficacy, only double-blind, randomized, controlled trials were included. For the evaluation of the safety, controlled, uncontrolled, and pre-clinical studies were included in the review. Concerning toxicity, none can be extrapolated to humans from in vitro results. In vivo studies do not identify any major toxicities. Concerning efficacy and safety, a total of 15 studies and 3 prospective clinical studies have been studied. For 1 g of fresh ginger root per day for four days, results show a significant decrease in nausea and vomiting and no risk for the mother or her future baby. The available evidence suggests that ginger is a safe and effective treatment for NVP. However, beyond the ginger quantity needed to be effective, ginger quality is important from the perspective of safety.

## 1. Introduction

According to the World Health Organization, the growth and expansion of traditional and complementary medicine (T&CM) products is worldwide phenomenon. These days, this sector plays a significant role in the economic development of number of countries [1]. The growing mistrust of the side effects of pharmaceuticals products coupled with the desire for more traditional medicines perceived as natural and safe by consumers, may partially explain the increase in the use of herbal remedies. 

In Europe, many herbal remedies are sold as food supplements and meet Directive 2002/46/EC [2]. Regarding botanicals, particularly, the European Food Safety Authority (EFSA) published, in 2012, a compendium of botanicals and associated substances of concern. The objective is to have a guideline for the evaluation of specific ingredients in food supplements, identifying the compound(s) to monitor [3]. In parallel, positive and/or negative lists of botanicals are published by authorities in different European countries [4,5,6,7] to control and guarantee the quality and traceability of used botanical ingredients in food supplements. These regulatory approaches aim to protect consumer health by ensuring that Complementary and Alternative Medicine (CAM) are safe and of high quality. 

More than 100 million Europeans use regularly CAM, and the prevalence varied from 5.9 to 48.3% [8]. However, women in the middle-age, tertiary educated are typical consumers. In France [9], women consume more food supplements than men. This is also true in Belgium [10], Australia, and the USA [11]. Despite higher consumption by women in Europe, there are variations by country [12]. The differences in consumption by country and gender are summarized in Table 1.

Not only women, but also pregnant women, consume food supplements. In the United States, 36.7% of pregnant women from the ages of 19 to 49 reported using CAM in the last year compared to 40.7% of non-pregnant women [13]. In the UK and Australia, 57.8% and 52% of pregnant women, respectively, had used an herbal remedy during pregnancy [14,15]. 

Some studies showed that the women had a positive opinion on the safety of herbal remedies during pregnancy [16,17]. Moreover, a minimum of one-third of the healthcare professionals are willing to recommend the use of CAM to pregnant women [18,19], and the majority (69.2%) agreed that there was some value in CAM use during pregnancy [20]. Nevertheless, the safety of CAM was also a key concern. Despite this, documentation on the safety and efficacy of many herbs used during pregnancy is limited. Whereas most Member States update and regularly implement regulations relative to herbal substances/products according to the most recent scientific evaluations, few toxicological data coming from studies on pregnant women are available. 

**Figure 1:**
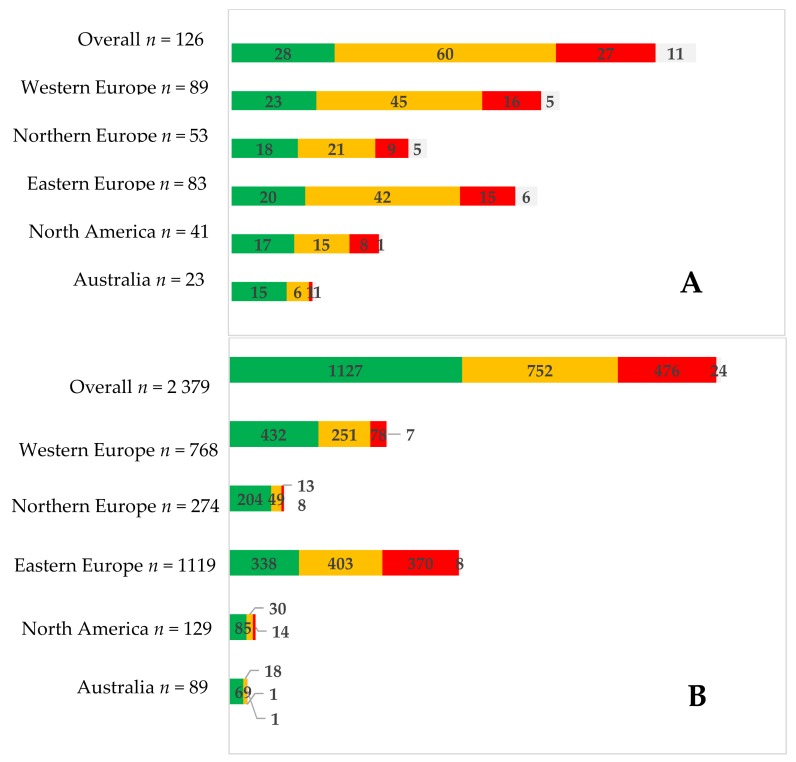
Herbal medicine used (**A**) and number of women who used them (**B**) by safety classification (green = safe; orange = caution; red = contraindicated; white = unknown). From Kennedy et al. [18].

A multinational, cross-sectional study [18] identified 126 specific herbal medicines used by 2379 women. They were classified on three categories: safe, caution, and contraindicated, and their consumption was observed. Women used herbal medicine mainly classified as safe for pregnancy use. However, a difference between regions was observed (Figure 1). This study shows that there is still work to be done in informing pregnant women. Information confirmed by Pallivalappila et al. [19], 61% (*n* = 127) of dietary supplement users during pregnancy and 44% (*n* = 54) of non-users responded that CAM should be available through the NHS (the publicly-funded healthcare system in Scotland).

One of the most popular botanical remedies during pregnancy is ginger (*Zingiber officinale* Roscoe) [14,18,21]. Ginger is an Asian native plant. Its aromatic rhizome is used as a spice, but also in traditional medicine since ancestral times. Ginger belongs to the official pharmacopoeias of different countries, including Austria, China, Egypt, India, United Kingdom, Japan, Switzerland, and the Netherlands.

For around 10 years, ginger imports in Europe have increased significantly [22], and many foods and food supplements have appeared on the market. Most of these food supplements are dedicated to pregnant women (Table 2).

Whereas ginger rhizome consumption is described for Nausea and Vomiting of Pregnancy (NVP) in different monographs [23,24,25], it is not recommended in others as a precautionary measure [26]. In the same way, ginger rhizome consumption by pregnant women is tolerated or authorized in several countries (France, Belgium) or forbidden in others (Finland, Russia). Without scientific evidence established in the concerned population, clear recommendations, and harmonized regulations, it is difficult for healthcare professionals to provide safe advice. 

This review focuses on the efficacy for NVP of ginger and the safety of its use in pregnant women. The main goal is to have an objective treatment of safety, so non-clinical and clinical data were analyzed to present and discuss factual information to healthcare professionals.

## 2. Generality

### 2.1. Zingiber Officinale Roscoe 

Ginger, *Zingiber officinale* Roscoe is originated from Asia. It is a plant of the genus *Zingiber* and of the family Zingiberaceae, whose rhizome is used worldwide in cooking and traditional medicine. It is considered as premium spices, like cardamom and turmeric. Ginger was first cultivated in the Asian subcontinent, probably in South East Asia [27]. It migrated to Europe by Greek and Roman times and was used as digestive aids wrapped in bread. Ginger was then incorporated into bread and confections. In the 1600s, the Spanish established ginger plantations in Jamaica and in the 19th century some physicians used it to induce sweating, to improve the appetite or decrease the nausea [28]. 

Ginger is cultivated in the tropical regions from both hemispheres and India is the largest producer (32.75% of the world’s production), followed by China (21.41%) and Nigeria (12.54%) [23,29].

### 2.2. Nutritional Composition and Chemical Composition

The main ginger constituents are starch (up to 50%), lipids (6 to 8%), proteins, and inorganic compounds [25,30]. When referring to the USDA National Nutrient Database for Standard Reference and DTU Fødevaredatabanken, raw ginger root is a source of potassium (415 mg/100 g). When referring to the ANSES Table Ciqual 2017 [31], data are very different: ginger powder is a source of phosphorus (168 mg/100 g) and is rich in magnesium (214 mg/100 g), potassium (1320 mg/100 g), manganese (33.3 mg/100 g), zinc (3.64 mg/100 g), iron (19.8 mg/100 g), and niacin (9.62 mg/100 g) (Table 3). These differences between nutrient databases showed high quantitative variations that could be explained by the plant variability itself but also by used analytical methods which can differ from database to another.

**Figure 2:**
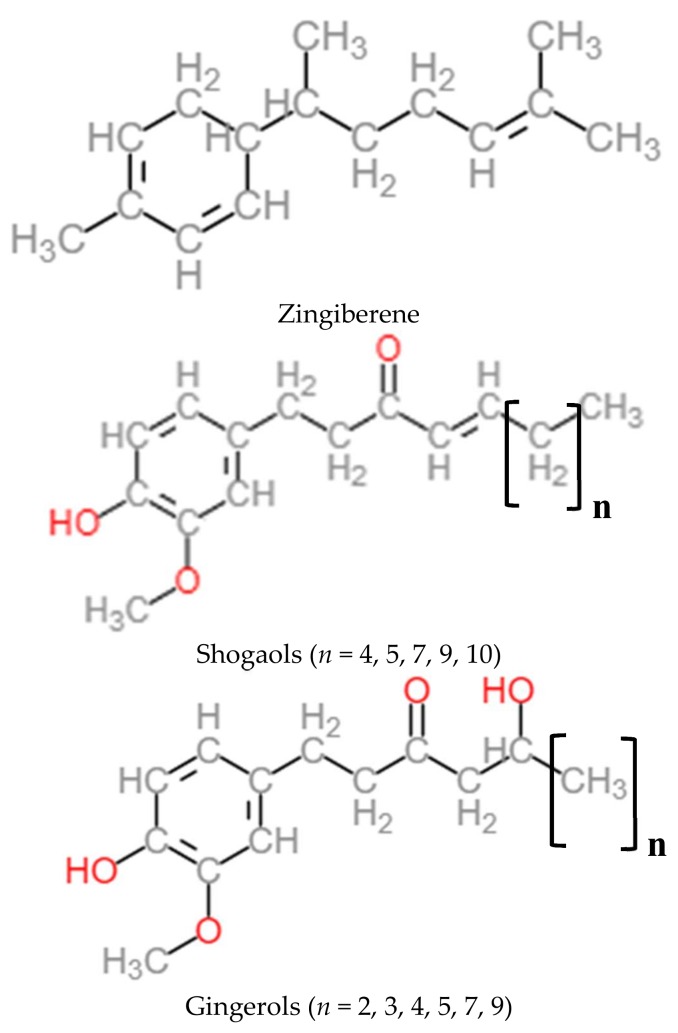
Chemical structures of active constituents: zingiberene, shogaols, and gingerols.

The non-volatile pungent principles responsible of spicy aroma are the gingerols, shogaols, paradols, and zingerone. They represent 4 to 7.5% [27,29] (Figure 2). The principal compound of these is 6-gingerol. In dried ginger, the concentrations of gingerols are reduced whereas the concentrations of shogaols are more abundant, coming from gingerol dehydration [27,32,33,34,35].

Volatile oils represent 1–4%. More than 100 compounds are identified. Most of them are terpenoids, mainly sesquiterpenoids (α-zingiberene, zingiberol, β-sesquiphellandrene…) and smaller amounts of monoterpenoids (camphene, cineole, geraniol…) [34,35].

All the *Z. officinale* samples coming from different origins are genetically indistinguishable. However, their metabolic profiling showed quantitative variation [27] function of the type, the variety but also the agronomic conditions, harvest, drying methods, and storage conditions [35,36].

### 2.3. Ginger Consumption 

In EU countries, the apparent consumption (henceforth referred to simply as ’consumption’) of ginger amounted to 58,000 tons in 2014 [22]. A large part of the consumption (around 70–80% of demand) comes from the food processing industry. Dried ginger is especially used in significant amounts in bakery (e.g., gingerbread, cookies) and Asian food products, as well as various drinks (e.g., ginger ale and ginger beer) [22].

Western EU countries accounted for 77% of EU consumption in 2014. The UK (32% of consumed volume), Germany (20%), and the Netherlands (13%) were the largest EU consumers. Concerning the UK, its large ethnic community is an important driver of the national consumption. The volume of consumed ginger in the EU increased by an average of 11% per year between 2010 and 2014, despite the economic crisis. This increase was greatest (over 20% per year) in the Eastern and Northern EU countries [22]. 

### 2.4. Women’s Perception of Ginger

In a multinational survey (Europe, North America, and Australia) including 9113 pregnant women and new mothers, Petersen et al. [37] determined their perception of risks related to medicines, foods or herbal substances, alcohol, tobacco, and thalidomide.

The least harmful products were cranberries and ginger, whereas antidepressants, alcohol, smoking, and thalidomide were rated as the most harmful. Mean risk perception scores for ginger is 1.5 on a 10-point scale (*n* = 8318, Table 4)

## 3. Non-Clinical Data

### 3.1. Cytotoxicity 

The cytotoxic potential of ginger has been studied for many decades. Various cell lines or in-tissue culture cells were used. Results appeared very different from one study to another (Table 5).

Wei et al. [43] indicated that diarylheptanoids and gingerol-related compounds were cytotoxic against human promyelocytic leukemia (HL-60) cells (IC50 < 50 μM), while Zaeoung et al. [42] reported that the IC50 of ginger was higher than 39.2 μg/mL against breast (MCF7) and colon (LS174T) cell lines. 

In 2008, Kim et al. [45] investigated the cytotoxicity of five compounds of ginger (4-, 6-, 8-, 10-gingerols, and 6-shogaol) on four human tumor cell lines. 6-shogaol showed the most potent cytotoxicity against the four tumor cell lines (IC50 from 1.05 to 1.76 μg/mL) while the others showed moderate activity [45]. Peng et al., for their part, isolated 13 compounds from fresh ginger and studied their cytotoxicity against nine human tumor cell lines. Three of them were identified to be cytotoxic in cell lines tested: 6-shogaol, 10-gingerol, and an enone-diarylheptanoid analog of curcumin [46].

When comparing the IC50 of different chemical constituents to their maximum plasma concentration (see Section 4.1: Pharmacokinetic Data) after an intake of 1.5 or 2 g of ginger, the IC50 value corresponds to concentrations that are not achievable in the human body. IC50 values represent from 7 to ca. 692 times the maximum concentration reached in the plasma (Table 6). 

Moreover, according to pharmacokinetics data [48] no free 6-, 8-, and 10-gingerols and 6-shogaol were detected in the plasma of all the volunteers 24 h after ginger consumption. Only glucuronide and sulfate metabolites were observed at very low concentration [48]. It is difficult to conclude that ginger is toxic in humans on the basis of these results. The studies presented here cannot be generalized to ginger activity in the body. The potential cytotoxic effects of ginger have to be studied, taking account of the whole plant and its absorption metabolites.

### 3.2. Genotoxicity/Mutagenicity 

Ginger showed some mutagenicity in TA 100, TA 1535 [49,50], and T 98 strains [41]. However, ginger’s mutagenicity was much lower than established mutagens, such as sodium azide or MNNG [49]. This mutagenicity could be linked to gingerols and shogaols [44,50]. However, in the presence of zingerone, mutagenic activity of gingerols and shogaols was suppressed in a dose-dependent manner (Table 7). 

Thus, the observed mutagenic activity of ginger extracts in vitro is the result of the combined action of pro and antimutagenic compounds present in the ginger [50]. However, as for cytotoxic assays, studies presented here cannot be extrapolated to in vivo mutagenicity because of the tested compounds and their concentrations, which are not representative to absorption metabolites and their blood concentration. 

Others in vitro and in vivo studies showed antimutagenic properties of ginger or ginger extracts [38,49,51]. When rats fed diets containing 0.5, 1, and 5% of powdered ginger for one month and are exposed to benzopyrene, the mutagenicity in vitro test realized with urine samples showed a reduced number of TA 98 and TA 100 revertants exposed to treated urine at all ginger concentrations compared to control urine [51]. On the basis of ingested dry matter, the ingested daily dose is approximately 0.1, 0.2, and 1 g of ginger by rats, which corresponds to 2.5, 5, and 25 g of ingested powdered ginger in humans, respectively. 

Plengsuriyakarn et al., using a crude ethanol extract of ginger, concluded there was an absence of any toxicity at maximum dose of 5 g/kg body weight (BW) using a hamster model [38].

In the in vitro microbial test system, ginger root has mutagenic and antimutagenic properties. In vivo, it appears to be antimutagenic when it is consumed at the human dose. 

### 3.3. Acute/Subacute Toxicity (Repeated Dose Toxicity) 

Many acute (oral administration of a single dose of a substance in rodents within 24 h) or subacute (oral administration of a test substance in rodents for 28 days) toxicity studies were realized with different rhizome ginger extracts or related compounds. Five studies with ginger powder or ginger extracts (freeze-dried or ethanol extracts) [38,52,53,54,55], and one with related compound [56] showed no treatment-related signs of toxicity or mortality in any animals at tested doses. One of them was realized on pregnant rats and showed neither embryotoxic nor teratogenic effects of the tested ginger [55]. The No Observable Adverse Effect Level (NOAEL) was 5000 mg/kg per day whatever the used extract (freeze dried or Ethanol extract) [38,53] whereas the NOAEL of ginger oil is up to 500 mg/kg per day [57]. In four studies [54,58,59,60], the oral LD50 is far above the highest dose used for human food, dietary supplements or drugs (Table 8). 

### 3.4. Reproductive Toxicity 

One in vivo study was made with a patented ginger extract [55]. Three groups of 22 pregnant female rats from days 6 to 15 of gestation received by gastric intubation of the extract in concentrations of 100, 333, and 1000 mg/kg and were killed on day 21 of gestation. A fourth group received sesame oil as control. Body weight, food, and water intakes were recorded during the study. Standard parameters of reproduction and performance were analyzed. Signs of teratogenic and fetuse toxic effects were determined. No adverse effects or deaths were observed and ginger supplementation was well tolerated. Neither embryotoxic nor teratogenic effects were showed by analysis of fetuses and the authors concluded that the ginger extract administered to pregnant rats during the organogenesis period caused neither maternal nor developmental toxicity at doses of up to 1 g/kg body weight per day [55].

By contrast, some ginger adverse events have been reported on pregnant rats [61]. Pregnant Sprague–Dawley rats received ginger tea (15, 20, or 50 g/L) on day 6 of gestation onwards until day 15 of gestation. They were sacrificed at day 20. No maternal toxicity was observed, but embryonic loss was increased in the treatment group compared to control. No gross morphologic malformations were seen but fetuses exposed to ginger tea were significantly heavier than control and had more advanced skeletal development. This effect was greater in female fetuses and not correlated with increased placental size. Thus, results of this study suggest that in utero exposure to ginger tea leads to an increased early embryo loss with increased growth in surviving fetuses [61]. 

In 2009, Sukandar et al. studied the effect of ethanol extracts of ginger and noni fruit in pregnant rats. The blend was administered per os at three different doses on days 6–15 of gestation (50 and 50, 500 and 500, 1000 and 1000 mg/kg BW). Sacrifices were realized on day 19 of gestation to observe live fetus, resorption, and growfail fetus. No malformation was found. The combination, did not cause any fetus resorption, growth failure, malformation in organs nor in the skeleton, whatever the dose. The highest dose (500 and 500 and 1000 and 1000 mg/kg BW) caused liver color change of 7.3% and 8.3% of rat fetuses, respectively [62]. 

In a double-blind randomized cross-over clinical trial, ginger per os, 250 mg, four times daily did not generate teratogenic aberrations in newborns and Apgar scores of these babies were 9 to 10 after five minutes [63]. 

Reproductive and developmental toxicity has been investigated in three studies in rats [55,61,62] and one double-blind randomized cross-over clinical trial [63]. All these studies showed no teratogenic aberrations. Extensive data do not suggest any major concerns with respect to reproductive and developmental safety of ginger root. 

## 4. General Population Safety Data 

Many reviews have been published on ginger effects and few minor adverse events have been reported with the use of ginger in humans [34,64,65,66,67].

A systematic review by Betz et al. [65] including 15 randomized studies with safety data showed that, among the 777 patients included, 3.3% reported slight side effects that did not require treatments like mild gastrointestinal symptoms and sleepiness.

In a clinical trial involving 12 healthy volunteers who consumed 400 mg of ginger three times per day for two weeks, one subject reported mild diarrhea during the first two days of treatment. Authors explained that ginger could cause heartburn and as a gastric irritant with doses higher than 6 g. Moreover, its inhalation could produce an IGE-mediated allergy [64]. 

An assessment report on *Zingiber officinale* Roscoe made by the Committee on Herbal Medicinal Products (HMPC) in 2012 summarized the published clinical studies with safety data indexed by PubMed through June 2010 [30]. 

Adverse effects resulting from the intake of ginger root observed in clinical studies occur with low frequency, low intensity, and are mainly gastrointestinal. No severe events have been reported. An interaction between ginger and warfarin has been showed in some case reports however the latter are unconvincing. Moreover, one randomized study in healthy volunteers failed to demonstrate any warfarin interaction. No sufficient evidence are available to suggest induction or inhibition of CYP-enzymes by ginger or its constituents [30]. Consequently, the committee concluded that the benefit/risk balance is in favor of the oral use of ginger extract and complies with the criteria for well-established use in the prevention of nausea and vomiting in motion sickness [30]. However, ginger’s effect on platelet aggregation cannot be confidently dismissed and future clinical trials are needed to further investigate this area, particularly in risk population [68].

More recently, Wang et al. [69] assessed daily ginger consumption in adults, explored its correlation with chronic diseases and analyzed further how different levels of ginger intake may affect the prevalence of chronic diseases. A total of 4628 participants (1823 men and 2805 women), aged from 18 to 77 years old, completed face-to-face dietary and health questionnaires. Daily ginger consumption was associated with a decreased risk for hypertension ((OR), 0.92; 95% confidence interval (CI), 0.86–0.98) and chronic heart disease (CHD) (OR, 0.87; 95% CI, 0.78–0.96) in adults ≥18 years old. Differences were also observed in adults ≥40 years old: hypertension (OR, 0.92; 95% CI, 0.87–0.99), CHD (OR, 0.87; 95% CI, 0.78–0.97). However, after 60 years old, no association was seen for hypertension, but there was still a difference regarding CHD (OR, 0.84; 95% CI, 0.73–0.96). Again, the probability of illness (hypertension or CHD) decreased when the dosage of daily ginger intake increased. No adverse events were reported [69].

### 4.1 Pharmacokinetic Data

In 2008, Zick et al. [47] realized a single dose pharmacokinetic escalation study of the ginger constituents 6-gingerol, 8-gingerol, 10-gingerol, and 6-shogaol. 27 healthy volunteers were recruited, three participants per dose except for the highest doses for which 6 and 9 participants were included. Administrated doses of ginger extract were 100 mg, 250 mg, 500 mg, 1.0 g, 1.5 g, and 2.0 g. The dry extract of ginger root used in the study was standardized to 6% of gingerols. A dose of 250 mg correspond to 15 mg of total gingerols, with 5.38 mg 6-gingerol, 1.28 mg 8-gingerol, 4.19 mg 10-gingerol, and 0.92 mg 6-shogaol. Blood was sampled at different times after ginger intake and analysis showed that no free 6-gingerol, 8-gingerol, 10-gingerol, or 6-shogaol was detected and no conjugate metabolite were detected below the 1 g ginger extract dose, except for 6-gingerol. However, as of this dose, all metabolites were quickly absorbed and detected as glucuronide and sulfate conjugates. Conjugate metabolite appeared 30 min after 2 g dose intake, with their T_max_ between 45 and 120 min and their elimination half-lives between 75 to 120 min. The maximum concentrations were 1.69 μg/mL for 6-gingerol, 0.23 μg/mL for 8-gingerol, 0.53 μg/mL for 10-gingerol, and 0.15 μg/mL for 6-shogaol at either the 1.5 g or 2.0 g dose. No pharmacokinetic model was able to be constructed due to the low levels of ginger constituent absorption, and the pharmacokinetic parameters were based on a non-compartment analysis with an elimination half-life only presented for the 2.0 g dose [47].

Another study made by Yu [48] in 2011 with 12 volunteers receiving 2 g of ginger extract per os for 24 days showed that no 6-, 8-, and 10-gingerols, and 6-shogaol under free form were detected in the plasma of all the participants 24 h after the last dosing. Concentrations of 6-gingerol glucuronide (from 5.43 to 13.6 ng/mL), 6-gingerol sulfate (from 6.19 to 7.29 ng/mL), and 10-gingerol glucuronide (from 6.96 to 9.33 ng/mL) were low whereas levels of other conjugate metabolites were not detectable in all the volunteers. Maximum concentrations determined for 10-gingerol and 6-shogaol were 9.5 ± 2.2 ng/mL (0.027 ± 0.006 μM) and 13.6 ± 6.9 ng/mL (0.049 ± 0.025 μM), whereas the IC50s of 10-gingerol and 6-shogaol were 12 and 8 μM, or 4.2 and 2.2 mg/mL, respectively. The half-lives of all metabolites are between 1 and 3h [48]. 

Table 6 reviews the pharmacokinetic data and IC50 values of some ginger metabolites absorbed and detected in plasma, such as 6-, 8-, 10- gingerols, and 6-shogaol. As mentioned before, IC50 values represents between 7 to 692 times the maximum concentrations detected in plasma. 

## 5. Pregnant Women Data

### 5.1. Safety Data

We analyzed 14 randomized clinical studies from 1991 [63] to 2017 [70] and three prospective studies [71,72,73]. In randomized clinical studies, a total of 1 331 pregnant women have been studied, including 617 who consumed ginger (Table 9 and Table 10). Paritakul et al. studied 63 postpartum women (33 for the placebo group and 30 for ginger group). Studies included pregnant women at less than 20 weeks of gestation and only for one greater than or equal to 37 weeks of gestation [74].

Different types of ginger were used, like fresh ginger, ginger powder, ginger extract, or ginger essence. The ginger dose ranges studied were from 500 mg/d to 2.5 g/d. The recommended daily doses were: 3 × 650 mg, 4 × 250 mg, 2 × 500 mg, 4 × 125 mg, 3 × 350 mg, 3 × 250 mg, and five biscuits. The majority of the studies were conducted over four days, but three studies chose seven days [75,76,77], one study was conducted for two weeks [78], and one study was conducted for three weeks [79]. Clinical studies were realized in Asia (Thailand), Europe (Denmark), Oceania (Australia), and the Middle East (Iran). The majority of studies (12/14) lasted between four and seven days, which is consistent with the symptomatology of pregnant women with regard to nausea and vomiting. Ten studies, all randomized versus placebo or positive control (vitamin B6 or dimenhydrinate), have highlighted various side effects. 

The various adverse effects founded in RCTs are listed in Table 11. There are no common side effects from one study to another with the exception of heartburn and spontaneous abortion, which are quoted six and five times, respectively. 

This diversity of side effects can be explained by changes in several parameters depending on the studies. The most important point is that ginger quality is never specified and there are many variations on the type of ginger, recommended daily doses, and the duration of treatment.

According to Boltman-Binkowski [87], ginger does not increase spontaneous abortion compared to the control group (relative risk with 95% CI = 0.80 (0.21, 2.99)) and does not increase the rate of stillbirth (relative risk with 95% CI = 0.64 (0.03, 13.59)) and congenital abnormalities (relative risk with 95% CI = 2.11 (0.07, 65.87)) compared to the general population.

In Saberi’s studies made in 2013 and 2014 [76,77], a total of 249 women participated to RCTs, 87 of whom are treated with ginger, and only two cases of heartburn were detected. Babies whose mothers were exposed to ginger during Saberi’s study did not appear to be at an increased risk of fetal abnormalities or low birth weight. All these side effects were reported by subjects as minor and did not preclude them from taking their prescribed medication [76,77]. 

Three prospective studies [71,72,73] focus on the exposure of pregnant women to ginger and the possible side effects that would result from it. In 69 361 women, 812 pregnant women were studied because they took ginger during first trimester. Choi et al. [73] noted seven cases of spontaneous abortions in the ginger group and 17 cases in the control group (OR: 0.8; 95% CI: 0.3–1.9; *p* = 0.59). At first reading, rates of stillbirths and babies that required attention at the neonatal intensive care unit (NICU) were marginally superior in the ginger group vs. the control group. However, no difference was observed when the NICU admission rate was compared with what was reported from the hospital and Women’s Healthcare Center in 2012.

Heitmann et al. [72] found that ginger use during pregnancy at any time did not increase the risk of malformations (4.7% in the no exposure to ginger group vs. 4,1% in those exposed to ginger during the first trimester group, adjusted OR (95 % CI) 0.8 (0.5–1.4)). Moreover, ginger use during pregnancy is not significantly associated to the risk of stillbirth/perinatal death, low birth weight, preterm birth, or low Apgar score. Equivalent results were found by Portnoi et al. [71], with no differences between the groups in terms of live births, spontaneous abortions, stillbirths, therapeutic abortions, birth weight, or gestational age. One exception: there were more infants who weighed less than 2500 g in the control group. Unfortunately, Heitmann et al. [72] specified neither the duration, nor the dose, nor the type of ginger used. Several types of ginger were consumed by women in Portnoi’s study [71] and Choi et al. [73] did not give any details except that it is a dried ginger.

Three studies [70,78,82], used ginger in syrup and powder form, respectively, on 14, 28, and 32 pregnant women during two weeks or four days. None of them had safety outcomes, but they concluded that ginger is a safe option in early pregnancy. 

Term birth parameter is mentioned in three others studies [79,80,83]. In two of them, there was no significant difference between ginger groups vs. other groups (placebo or B6) [80,83]. Conversely, Smith, et al., showed that term birth in the ginger group was significantly better than placebo (93% vs. 98% for placebo and of ginger groups, respectively, *p* = 0.03) [79].

A randomized, double-blind controlled trial was conducted by Paritakul et al. [74] in 2016 on women for seven days postpartum. Sixty-three women (30 ginger group, 33 placebo group) received 500 mg of dried ginger powder in capsules twice a day and no notable side effects are mentioned. 

The 14 randomized and three prospective clinical studies have observed more than 70,000 pregnant women, including more than 1500 who have consumed ginger during their pregnancy. Studies ran through over more than 25 years. Doses, durations, types of ginger, and countries vary from one study to another, thus addressing the different possibilities of current consumption. The use of ginger during pregnancy do not present a risk for the mother or her future baby. All studies conclude the safety of ginger. We can, however, note a side effect inherent to the composition of ginger—heartburn—which must be monitored in sensitive persons. 

According to McLay [88], ginger could potentially cause interactions with concurrent prescription medicines: one major interaction with nifedipine, and three moderate interactions with metformin, insulin, and aspirin. In France, these drugs—except insulin—are not recommended for use or are contraindicated in pregnant women. 

### 5.2. Efficacy 

The mechanisms of action underlying ginger’s efficiency in reducing NVP has been investigated [89], dual antiemetic action have been highlighted: (i) gingerols and shogaols act as antagonists of cholinergic M3 and serotonin 5-HT3 receptors of the central nervous system; (ii) ginger’s constituents improve the gastric tonus, motility, and emptying due to peripheral anticholinenergic and antiserotonergic actions. However, these data need further investigations to elucidate and confirm those preliminary findings.

Concerning the efficacy of ginger on pregnant women, Mohammadbeigi‘s study is added to the previous studies mentioned in Section 5.1 [84]. The total number of pregnant women studied in randomized clinical studies was 1433, with 651 pregnant women who consumed ginger. The doses used and the duration of treatment vary according to the studies. 

To determine the effectiveness of ginger on NVP, its effect was compared to control [77] or placebo [77,78,80,84,85,86] groups, but also vitamin B6 [70,79,81,83], drugs like dimenhydrinate [75], metoclopramide [84], or acupuncture [76]. 

A minimum of 1 g of fresh ginger root per day during at least four days significantly decreased nausea and vomiting and improved significantly symptoms vs. placebo or vitamin B6 [79,80,81,83].

Willets, et al., [85] investigated in a randomized double-blind placebo-controlled study, the effect of a ginger extract (EV.EXT35) on morning sickness symptoms in 120 pregnant women. 125 mg of ginger extract was equivalent to 1.5 g of dried ginger. After four days, the nausea experience score was significantly less than zero (except for day 3). Concerning vomiting symptoms, there was no significant difference between ginger extract and placebo groups

Keating et al. [78] demonstrated that 1 g/d (4 × 250 mg) of ginger syrup for a two-week period revealed that 67% of women in the ginger group stopped vomiting at day 6 vs. 20% in the placebo group. Additionally, 77% of women in the ginger group had a four-point improvement on the nausea scale at day 9 vs. 20% in the placebo group.

Mohammadbeigi et al. [84] used 600 mg/d (3 × 200 mg) of ginger essence for five days vs. 30 mg/d (3 × 10 mg) of metoclopramide and 600 mg/d (3 × 200 mg) of placebo. Significant decreases in the severity of nausea and vomiting, as well as the Rhodes Index for ginger and metoclopramide groups vs. the placebo group, was shown. There was also no difference between both groups. 

Basirat et al. [86] realized a randomized double-blind clinical trial on 62 pregnant women. Thirty-two women took five biscuits daily for four days. Each biscuit contained 0.5 g of ginger. The average change in nausea scores in the ginger group was significantly better (*p* = 0.01) than that in the placebo group. The average change in the number of vomiting episodes was not significantly different between the ginger group and the placebo group. However, after four days of treatment, the proportion of women who had no vomiting in the ginger group (11/32 patients) was greater than that in the placebo group (6/30). 

Regardless of the dose, duration, or type of ginger, there is a significant impact on nausea and there are no safety concerns. In 4/15 studies either ginger had no effect on vomiting or there was no significant difference with placebo or vitamin B6.

According to Ding et al. [90], all various forms of ginger studied were a safe and effective treatment for NVP when compared to placebo and vitamin B6. Two meta-analyses made in 2014 [67,91] concluded also that ginger could be considered as an effective and harmless alternative option for women suffering from the symptoms of NVP and that 1 g/day for a duration of at least four days is better than placebo in improving NVP.

## 6. Conclusions 

NVP affect 7 in 10 pregnant women and have a deep impact on the quality of life. Recently, increasing concerns have been pointed out regarding the safety of traditional antiemetic drugs (metoclopramide, domperidone, etc.). Thus, considering natural options, such as ginger, with a favorable risk/benefit ratio and a good level of evidence, is now part of several practice guidelines [92].

Many food supplements with ginger powder or ginger extracts are used to decrease symptoms of nausea and vomiting associated with pregnancy. This effect is supported by a European claim (ID 2172) that it “*Helps to support the digestion/contributes to the normal function of intestinal tract/contributes to physical well-being/contributes to the normal functioning of the stomach in case of early pregnancy*” provided the product contains the equivalent of 0.5 to 2 g of root per day.

Beyond the quantity of ginger needed to be effective, the quality of the ginger is important for the safety aspect. For example, in France, it is mandatory to monitor the concentration of methyleugenol because of its potential toxicity [5]. It is important to focus special attention on pungent components of ginger powder, including the main components, 6-gingerol, 8-gingerol, and 10-gingerol [89]. It is also important to highlight the wide variability in 6-gingerol, 6-shogaol, 8-gingerol, and 10-gingerol composition from one food supplement to another. The 6-gingerol concentration ranged from 0.0 to 9.43 mg/g, 6-shogaol ranged from 0.16 to 2.18 mg/g, 8-gingerol ranged from 0.0 to 1.1 mg/g, and 10-gingerol ranged from 0.0 to 1.40 mg/g [93]. Variations could be due to sourcing, method and period of harvest, storage, and processing methods [93]. The main critical control points to assure safety and quality of ginger used are chemical constituents (actives like gingerols, or potentially toxics like methyleugenol), contaminants (microbiology, pesticides, heavy metals, residual solvents), and adulteration risks. In view of the sensitivity of this raw material, control and qualification procedures appear mandatory: supply chain transparency, traceability, management of material safety, and quality standards are keys to assure consumer safety. Processes have to be in place to approve the suppliers’ production sites and the relationship between buyer and supplier is critical to support any adulteration prevention effort. 

With regard to toxicity associated with ginger chemical constituents, such as gingerols, cytotoxic or mutagenic in vitro studies already performed are not representative and difficult to extrapolate to humans. Potential toxic effect of ginger have to be studied taking account of the whole plant and its absorption metabolites in humans (glucuronides and sulfates of gingerols, for example), not plant metabolites. Recently, one in vitro study showed that free forms are more cytotoxic compared to the glucuronide conjugates [94]. 

All the in vivo results do not suggest any major concerns with respect to reproductive and developmental safety of ginger root. At least, no associations were found between the use of ginger and malformations in humans. This finding is reassuring and supports previous findings. Moreover, according to the data, the use of ginger during pregnancy does not increase the risk for any of the following pregnancy outcomes: stillbirth/perinatal death, low birth weight, preterm birth, and low Apgar score [72]. 

It is interesting to note that one member of the Committee on Herbal Medicinal Products (HMPC) did not agree with HMPC’s opinion on *Zingiber officinale* Roscoe rhizome [30,95]. He reported that ginger is used in food without any restrictions. Additionally, there are results of tests on reproductive toxicity and results of clinical trials including pregnant women published which support safe use during pregnancy. Therefore, the restriction for use during pregnancy is not justified. Moreover the results of the clinical trials for pregnancy-induced vomiting are such a quality that well-established use could be established for ginger rhizomes [92].

Finally, a recent consensus [96] was published on a list of benefits and potential harms of ginger use for the management of NVP. The authors suggest that this guideline should be addressed during the clinical consultation for NVP and should help to take a decision to use ginger or not. They reported that even if no conclusive evidence of adverse events of ginger on fetus was shown nowadays, the potential anti-coagulant effect of ginger is still equivocal and have to take account by clinicians before ginger recommendation. 

Can we safely give ginger rhizome to decrease nausea and vomiting in women during early pregnancy? 

First, medical supervision is mandatory. Ginger recommendation has to be done on a case-by-case basis after evaluation of patient medical history. Then, the quality of the finish product containing ginger and the quality of ginger itself, the quality of its transformation process (powder, extract, oils, etc.), and its relative standardization have to be mastered to assure consumer safety. If all the prerogatives are met, doctors could recommend ginger for NVP in early pregnancy.

At the least, further clinical studies are still needed to highlight the effect of ginger on platelet aggregation, particularly in pregnant women population. 

## Figures and Tables

**Table 1:** Comparison of food supplement consumption between men and women by country [12].

Country	Men (%)	Women (%)
France	17–21.6	26.3–28.5
Belgium	11–14	22.5–30
Australia	34.9	50.3
USA	45	58
Greece	2	6.7
Spain	5.9	12.1
Italy	6.8	12.6
Germany	20.7	27
The Netherlands	16	32.1
UK	36.3	47.5
Denmark	51	65.8
Sweden	30.5	42.4

**Table 2:** Sample of food supplements with ginger sold around the world; composition and claims.

Country	Composition	Daily Dose	Claims
Belgium	Cellulose; standardized ginger extract 50 mg with 10% gingerols (equal to 500 mg of powder); calcium phosphate; silicon dioxide, magnesium stearate; hypromellose, titanium dioxide; talc; glycerol	2/day for pregnant women	Helps you regain optimal digestive balance in the following situations: - in case of overeating - in case of unusual diets - when agitated or on trips - too much stress Can be used in pregnant women who wish to return to digestive well-being during pregnancy
	Ginger bio extract (6 × concentrated) 200 mg	1/day	Digestive comfort
	500 mg of ginger rhizome powder and 5 mg of ginger rhizome extract per caps	1/day	Sexual fatigue; nausea in pregnant women; travel sickness
	Ginger rhizome extract: 67 mg, eq. to 1 g of ginger rhizome, magnesium carbonate, vitamin B6	1/day	Ginger contributes to the normal functioning of the stomach in case of early pregnancy (Claim under consideration (EFSA)) Magnesium contributes to a reduction of tiredness and fatigue Vitamin B6 contributes to the regulation of hormonal activity
France	Organic ginger rhizome powder: 250 mg	4/day	Nausea in pregnant women
	Ginger rhizome powder (*Zingiber officinale* R): 365 mg/hard caps	4/day	Lower bowel contractions and digestive acids; prevent motion-induced nausea and vomiting
	Organic ginger rhizome extract (*Zingiber officinale* R): 200 mg	2/day	Ginger contributes to the normal functioning of the stomach in case of early pregnancy
	Organic ginger rhizome extract (*Zingiber officinale* R): 200 mg	5/day	Travel sickness
	*Zingiber officinalis* R (roots): 230 mg.	6/day	Helps to support the digestion/contributes to the normal function of intestinal tract Travel sickness Joint mobility Libido in men
	Sorbitol, rhizome ginger extract (*Zingiber officinale* R), natural orange flavor, sodium starch glycolate, microcrystalline cellulose, natural lemon flavor, magnesium stearate, silica, sucralose.	2/day	-
	Ginger, lemon, B6, magnesium, iron, B9	5 biscuits/day	-
	B6, folates, cocoa, strawberry pulp, ginger	1/day	-
	Ginger (*Zingiber officinale* Roscoe), cinnamon (*Cinnamomum verum* J. Presl), fennel (*Foeniculum vulgare* Mill.), lemon (*Citrus limon* (L.) Burm f.), Tumeric (*Curcuma longa* L.), hibiscus (*Hibiscus sabdariffa* L.), liquorice (*Glycyrrhiza glabra* L), natural lemon flavor and natural vanilla flavor	1–3 sachets /day	-
	Cellulose, silicon dioxide, magnesium stearate, standardized ginger extract (*Zingiber officinale* R) 50 mg; HPMC, titanium dioxide, talc, copper complexes of chlorophyllins	Pregnant women: 2/day Children from 6 to 11 years old: 1 to 4/day Adults and Children >12 years old: 2 to 8/day	Nausea in pregnant women; travel sickness Ginger contributes to the normal functioning of the stomach (Claim under consideration (EFSA))
	Cellulose, standardized ginger extract (10% of gingerols) 50 mg eq. to 500 mg of ginger powder; calcium phosphate; silicon dioxide; magnesium stearate; HMPC; titanium dioxide, talc, glycerol.	2/day for pregnant women	Helps to support the digestion/contributes to the normal function of intestinal tract/contributes to the normal functioning of the stomach in case of early pregnancy or travel sickness For adults and children aged 12 years and above
	Microcrystalline cellulose, standardized ginger extract 50 mg, eq. to 500 mg of rhizome powder, silicon dioxide, fatty acid magnesium salts, hypromellose, titanium dioxide, talc, glycerol	2 hard caps 3 times per day	Helps to support the digestion Contributes to decrease discomforts in case of travel nausea, pregnant women nausea, or chemotherapy
	Bach flowers, essential oils, and plant extracts	5 sprays if nauseous	Quick-acting oral spray against discomfort, apprehension and transport.
	Ginger root extract (160 mg of extract, 16 mg of gingerols); lemon balm extract (100 mg)	4/day	Stomach; antiemetic; pregnant women
Italy	Standardized root extract: 300 mg (5% gingerols) Whole root pulverized: 1500 mg	2/day	Regulate the gastro-intestinal motility and gas elimination of in case of nausea Promotes joint function and counteracts the localized states of tension Counter menstrual cycle disorders
	Hydro-alcoholic standardized ginger extract (5% of gingerols)	2/day	Reducing nausea, gas, bloating and intestinal spasms
Poland	Ginger rhizome extract 75 mg (eq. to 300 mg of powder); Vitamin B6: 0.5 mg	3/day	Adults and children poorly tolerating travel by vehicles and craft: cars, buses, trains, ships, planes
UK	Ginger extract 55 mg eq. to 1.1 g of root powder	1/day	Helps calm a queasy stomach; an excellent stomach soother; excellent travel companion
	Ginger extract 120 mg eq. to 14 g of root powder (24 mg of gingerols) (120:1 extract)	1/day	Do not take if pregnant or breastfeeding
	Ginger extract 138 mg eq. to 550 mg	2/day	-
	Ginger root 500 mg	2/day	-
	Ginger root extract (*Zingiber officinale* R) 500 mg, vitamin B6 25 mg, vitamin D 200 IU, calcium (carbonate and lactate) 240 mg, red raspberry leaf 25 mg, mint leaf 25 mg *Citrus aurantifolia* 10 mg Contains no artificial colors or flavors.	2/day	-
US	Ginger root powder	2/day	-
	Ginger root powder	2/day	May soothe an upset stomach and support digestion Promotes a healthy inflammation response May help reduce nausea, vomiting and dizziness
	Ginger root powder	2/day	Helps calm a queasy tummy an excellent stomach soother Excellent travel companion

**Table 3:** Nutritional composition of ginger root (USDA, ANSES, DTU).

	USDA (National Nutrient Database for Standard Reference)	ANSES (Table Ciqual 2016 Composition Nutritionnelle des Aliments)	DTU (Fødevaredatabanken Version 7.01)
Ginger type	Ginger Root, Raw	Ginger, Powder	Ginger Root, Raw
Phosphorus (mg/100 g)	34	168	34
Magnesium (mg/100 g)	43	214	43
Potassium (mg/100 g)	415	1 320	415
Manganese (mg/100 g)	-	33.3	0.229
Zinc (mg/100 g)	0.34	3.64	0.34
Iron (mg/100 g)	0.60	19.8	0.60
Calcium (mg/100 g)	16	114	16
Niacin (mg/100 g)	0.750	9.62	0.950
Folate (μg/100 g)	11	13	11

**Table 4:** Mean risk perception scores for 13 individual items by geographical region (dark green = 0–2; pale green = 2–4; yellow = 4–6; orange = 6–8; red = 8–10). From Petersen et al. [37].

	Australia	Eastern Europe	Eastern Europe	Western Europe	North America
Cranberries					
Ginger					
Eggs					
Paracetamol					
OTC (against nausea					
Antibiotics					
Swine flu vaccine					
Blue-veined cheese					
Dental X-ray					
Antidepressants					
Alcohol (1st trimester)					
Smoking					
Thalidomide					

**Table 5:** In vitro cytotoxicity of ginger extracts and main related compounds.

Ginger, Ginger Extracts, or Related Compounds	Cell Line	IC50	Reference
Ethanol extract of ginger	CL-6–Calcein assay	10.95 µg/mL	Plengsuriyakarn et al., 2012 [38]
Ethanol extract of ginger	CL-6–Hoechst 33342 assay	53.13 µg/mL	Plengsuriyakarn et al., 2012 [38]
Ethanol extract of ginger	HepG2–Calcein assay	71.89 µg/mL	Plengsuriyakarn et al., 2012 [38]
Ethanol extract of ginger	HepG2–Hoechst 33342 assay	92.88 µg/mL	Plengsuriyakarn et al., 2012 [38]
Ethanol extract of ginger	HepG2	358.71 µg/mL	Harliansyah et al., 2007 [39]
Ethanol extract of ginger	HRE–Calcein assay	198.15 µg/mL	Plengsuriyakarn et al., 2012 [38]
Ethanol extract of ginger	HRE–Hoechst 33342 assay	245.91 µg/mL	Plengsuriyakarn et al., 2012 [38]
Ethanol extract of ginger	Hamster ovary	245 µg/mL	Unnikrishnan et al., 1988 [40]
Ethanol extract of ginger	Vero cells	120 µg/mL	Unnikrishnan et al., 1988 [40]
Ethanol extract of ginger	Dalton’s Lymphoma Ascites	200 µg/mL	Unnikrishnan et al., 1988 [40]
Aqueous extract of ginger	Dalton’s Lymphoma Ascites	420 µg/mL	Unnikrishnan et al., 1988 [40]
Aqueous extract of ginger	NRK-52E Cells (MTT test)	No cytotoxicity	Abudayyak et al., 2015 [41]
Chloroform extract of ginger	NRK-52E Cells (MTT test)	9.08 mg/mL	Abudayyak et al., 2015 [41]
Methanol ginger extract	MCF7 (Breast)	75 µg/mL	Zaeoung et al., 2005 [42]
Methanol ginger extract	LS174T (Colon)	80 µg/mL	Zaeoung et al., 2005 [42]
Volatile oil of ginger	MCF7 (Breast)	14.2 µg/mL	Zaeoung et al., 2005 [42]
Volatile oil of ginger	LS174T (Colon)	15.9 µg/mL	Zaeoung et al., 2005 [42]
**Related compounds**			
Diarylheptanoids and gingerol-related compounds	HL-60	<50 µmol/L	Wei et al., 2005 [43]
6-gingerol	HepG2	431.7 µg/mL	Harliansyah et al., 2007 [39]
6-gingerol	MCF7 (Breast)	31.6 µg/mL	Zaeoung et al., 2005 [42]
6-gingerol	LS174T (Colon)	30.6 µg/mL	Zaeoung et al., 2005 [42]
6-gingerol	HepG2	89.58 µg/mL	Yang et al., 2010 [44]
6-shogaol	MCF7 (Breast)	6 µg/mL	Zaeoung et al., 2005 [42]
6-shogaol	LS174T (Colon)	4.2 µg/mL	Zaeoung et al., 2005 [42]

**Table 6:** IC50 values and plasma concentration comparison.

Ginger Related-Compounds	IC50 (µg/mL)	Plasma Concentration after 1.5 or 2 g of Ginger (µg/mL)	Ratio IC50/Concentration	References
6-gingerol	15.72 to 431.7	1.69	9.3 to 255.4	Zaeoung et al., 2005 [42]; Harliansyah et al., 2007 [39]; Kim et al., 2008 [45]; Zick et al., 2008 [47]
6-shogaol	1.05 to 6	0.0136 to 0.15	7 to 441.2	Zaeoung et al., 2005 [42]; Kim et al., 2008 [45]; Zick et al., 2008 [47]; Yu et al., 2011 [48]
8-gingerol	8.85 to 12.57	0.23	38.5 to 54.7	Kim et al., 2008 [45]; Zick et al., 2008 [47]
10-gingerol	4.2 to 6.57	0.0095 to 0.53	7.9 to 691.6	Kim et al., 2008 [45]; Zick et al., 2008 [47]; Yu et al., 2011 [48]

**Table 7:** Genotoxicity/mutagenicity of ginger and related compounds.

	**Test Object**	**Concentration**	**Results**	**References**
**In Vitro**				
**Ginger**				
Ames assay	*Salmonella typhimurium* strains TA 100, TA 98, TA 1535, and TA 1538 with and without activation	5 to 200 µg/plate	Mutagenic on metabolic activation in strains TA 100 and TA 1535	Nagabhushan et al., 1987 [50]
Ames assay	*S. typhimurium* TA 100 and TA 1535, with and without activation	25 and 50 mg/mL	Mutagenic in both TA, at both concentrations	Soudamini et al., 1995 [49]
Ames assay	*S. typhimurium* TA 100 and TA 98, with and without activation	0.78 to 25 mg/mL	Mutagenic on TA 98 activated cells from 3.13 mg/mL	Abudayyak et al., 2015 [41]
**6-gingerol**				
Comet assay	HepG2	0 to 80 µmol/L	DNA strand breaks from 5.89 mg/mL (20 µmol/L)	Yang et al., 2010 [44]
Ames assay	*Salmonella typhimurium* strains TA 100, TA 98, TA 1535, and TA 1538 with and without activation	5 to 200 µg/plate	Mutagenic on metabolic activation in strains TA 100 and TA 1535	Nagabhushan et al., 1987 [50]
**Shogaol**				
Ames assay	*Salmonella typhimurium* strains TA 100, TA 98, TA 1535, and TA 1538 with and without activation	5 to 200 µg/plate	Mutagenic on metabolic activation in strains TA 100 and TA 1535	Nagabhushan et al., 1987 [50]
**Zingerone**				
Ames assay	*Salmonella typhimurium* strains TA 100, TA 98, TA 1535, and TA 1538 with and without activation	5 to 200 µg/plate	No mutagenic effect	Nagabhushan et al., 1987 [50]

**Table 8:** Acute/subacute toxicity of ginger extracts and the main related compounds.

	**Administration**	**Species**	**Oral LD50**	**References**
**Ginger dried rhizome powder**				
Ginger powder	per os	Rats	No mortality	Rong et al., 2009 [52]
**Ginger dried rhizomes extracts**				
Ginger dried rhizomes ethanol extract	intraperitoneal	Mice	1551 ± 75 mg/kg	Ojewole et al., 2006 [58]
Ginger dried rhizomes ethanol extract	per os	Hamsters	No mortality	Plengsuriyakarn et al., 2012 [38]
Patented standardized ethanol extract of dried rhizomes of ginger (EV.EXT 33)	per os	Pregnant rats	No mortality	Weidner et al., 2001 [55]
Freeze-dried ginger powder	per os	Rats	No mortality	Malik et al., 2011 [53]
Dry ginger decoction	per os	Rats: Gastric ulcer models	250 g/kg	Wu et al., 1990 [60]
Roasted ginger decoction	per os	Rats: Gastric ulcer models	170.6 g/kg	Wu et al., 1990 [60]
Ginger dried rhizomes ethanol extract		Mice	No mortality at 2.5 g/kg	Anonymous, 2003 [54]
Ginger oil	per os	Rabbits	5 g/kg	Anonymous, 2003 [54]
Ginger oil	per os	Rats	No mortality	Jeena et al., 2011 [57]
**Related compounds**				
**(E)-8 beta,17-epoxylabd-12-ene-15,16-dial (ZT)**				
(E)-8 beta,17-epoxylabd-12-ene-15,16-dial (ZT) from ginger	per os (25 mg/kg)	Mice	No mortality	Tanabe et al., 1993 [56]
(E)-8 beta,17-epoxylabd-12-ene-15,16-dial (ZT) from ginger	Intra-abdominal (25 mg/kg)	Mice	No mortality	Tanabe et al., 1993 [56]
**6-shogaol**				
6-shogaol	intravenous	Mice	50.9 mg/kg	Suekawa et al., 1984 [59]
6-shogaol	intraperitoneal	Mice	109.2 mg/kg	Suekawa et al., 1984 [59]
6-shogaol	per os	Mice	687 mg/kg	Suekawa et al., 1984 [59]
**6-gingerol**				
6-gingerol	intravenous	Mice	25.5 mg/kg	Suekawa et al., 1984 [59]
6-gingerol	intraperitoneal	Mice	58.1 mg/kg	Suekawa et al., 1984 [59]
6-gingerol	per os	Mice	250 mg/kg	Suekawa et al., 1984 [59]

**Table 9:** Randomized double-blind trials investigating the effectiveness and safety of ginger for pregnancy.

Objective	Population	Number	Treatment	Ginger Definition	Duration	Results	Adverse Events	Reference
To determine the effectiveness of ginger for the treatment of NVP	Women NVP < 17 weeks of gestation	*n* = 67 35 placebo 32 ginger	1000 mg/day (4 × 250 mg) of ginger (powder capsules) vs. placebo	Fresh ginger root	4 days + follow-up visit 7 days later	Significant decrease of nausea in the ginger group vs. placebo group (*p* = 0.014) Significant decrease of vomiting in the ginger group vs. placebo group (*p* < 0.001) Follow-up visits:Significant symptom improvement in the ginger group vs. placebo group (*p* < 0.001)	Headache: - 5 in the placebo group - 6 in the ginger group. Ginger group: - 1 abdominal discomfort - 1 heartburn - 1 diarrhoea for one day **Side effects reported as minor.** Spontaneous abortions: - 3 in the placebo group - 1 in the ginger group Term delivery: - 91.4% in the placebo group - 96.9% in the ginger group Cesarean deliveries: - 4 in the placebo group - 6 in the ginger group **No infants had any congenital anomalies recognized.** **No significant adverse effect of ginger on pregnancy outcome was reported.**	Vutyavanich et al., 2001 [80]
To compare the effectiveness of ginger and vitamin B6 for treatment of NVP	Women NVP < 16 weeks of gestation	*n* = 123 62 vit. B6 61 ginger	1950 mg/day of ginger (3 × 650 mg) or 75 mg/day of vitamin B6 (3 × 25 mg)	Fresh ginger root	4 days	Nausea/vomiting: Improvement of nausea vomiting scores in both group from baseline The average score change in the ginger group was better than that of vitamin B6 group (*p* < 0.05)	Side effects: - 16 in ginger group - 15 in B6 group (NS) Heartburn: - 8 in ginger group - 2 in B6 group Sedation: - 7 in ginger group - 11 in B6 group Arrhythmia: - 1 in ginger group Headache: - 2 in B6 group **Side effects were reported to be minor**	Chittumma et al., 2007 [81]
To determine the effectiveness of ginger for the treatment of NVP	Women NVP < 20 weeks of gestation	*n* = 67 35 placebo 32 ginger	1000 mg/day (4 × 250 mg) of ginger (powder capsules)	Ginger root powder (Zintoma, Goldaroo Company, Tehran, Iran)	4 days	Nausea: Significant improvement in 84% of ginger users vs. 56% in the control group (*p* < 0.05) Vomiting: Significant improvement in the treated group vs. the control group (*p* < 0.05)	No complication during the treatment period was reported **Reported as a safe remedy to improve the nausea and vomiting of pregnancy.**	Ozgoli et al., 2009 [82]
To compare the effectiveness of ginger and vitamin B6 for the treatment of NVP	Women with nausea with or without vomiting < 17 weeks of gestation	*n* = 69 34 vit. B6 35 ginger	1000 mg/day (2 × 500 mg) of ginger (powder capsules) or 40 mg/day of vitamin B6 (2 × 20 mg)	Fresh ginger root	4 days + follow-up visit 7 days later	Nausea score: Better score in the ginger group vs. the vitamin group (*p* = 0.024) Vomiting episodes: No significant difference between the groups Follow-up visits: 82.8% reported an improvement in the ginger group vs. 67.6% in the vitamin group (*p* = 0.52)	Spontaneous abortions: - 2 in the ginger group - 1 in the B6 group (*p* > 0.05) Term birth: - 82.9% in the ginger group - 82.4% in the B6 group Caesarean deliveries: - 4 in the ginger group - 6 in the B6 group (*p* > 0.05) **No babies had any congenital anomalies All were discharged in good condition** **No adverse effects of ginger on pregnancy outcome were reported**	Ensiyeh et al., 2009 [83]
To compare the effects of ginger on nausea and vomiting caused by pregnancy and compares it with metoclopramide medicine	Women NVP < 20 weeks of gestation	*n* = 102 34 placebo 34 metoclopramide 34 ginger	600 mg/day (3 × 200 mg) ginger;30 mg/day (3 × 10 mg) metoclopramide;600 mg/day (3 × 200 mg) placebo	Ginger essence	5 days	Intensity of nausea: Significant difference in the two supplemented groups (ginger or metoclopramide) vs. placebo (*p* < 0.05) Not statistically significant between treated groups	-	Mohammadbeigi et al., 2011 [84]
To determine if ginger syrup mixed in water is an effective remedy for the relief of NVP	Women with nausea with or without vomiting first trimester	*n* = 23 10 placebo 13 ginger	1 g/day (4 × 250 mg) ginger (tablespoon) vs placebo	Ginger including 1 mg pungent compounds from ginger rhizome juice, 1 mg of 20% pungent compounds and 5% zingiberene coming from CO_2_ supercritical extract of ginger rhizome	2 weeks	Nausea: 77% improvement in ginger group vs. 20% in placebo group Vomiting: 67% in ginger group stop vomiting at day 6 vs 20% in placebo group	Delivered viable infant at term without major complications **Safe option in the treatment of NVP**	Keating et al., 2002 [78]
To investigate the effect of a ginger extract (EV.EXT35) on the symptoms of morning sickness.	Women with morning sickness < 20 weeks of gestation	*n* = 9951 placebo48 ginger	500 mg/day (4 × 125 mg) eq. 6 g/day ginger vs. placebo	EV.EXT35: ginger extract (125 mg eq to 1.5 g of dried ginger)	4 days	Nausea experience score: - except for day 3, the difference parameter for each day post-baseline, was significantly less than zero Vomiting: - no significant difference between the groups	Spontaneous abortion: - 3 in the ginger group - 1 in the placebo group Intolerance of the treatment: - 4 in the ginger group Worsening of treatment requiring further medical assistance: - 1 in the ginger group - 2 in the placebo group Allergic reaction to treatment: - 1 ginger group **No apparent increased risk of fetal abnormalities or low birth weight.**	Willetts et al., 2003 [85]
To estimate whether the use of ginger to treat nausea or vomiting in pregnancy is equivalent to pyridoxine hydrochloride (vitamin B6)	Women NVP Between 8 and 16 weeks of gestation	*n* = 235 115 vit. B6 120 ginger	1.05 g/day ginger (3 × 350 mg) vs. 75 mg/day vitamin B6 (3 × 25 mg)	-	3 weeks	53% reported an improvement taking ginger, and 55% reported an improvement with vitamin B6 Ginger was equivalent to vitamin B6 for improving nausea, dry retching, and vomiting	Belching: - ginger (9%) vs. B6 (0%) (*p* < 0.05) Dry retching after swallowing: - ginger (52%) vs. B6 (56%) Vomiting after ingestion: - ginger (2%) vs. B6 (1%) Burning sensation: - ginger (2%) vs. B6 (2%) Pregnancy outcome: - 272 (93%) gave birth to 278 infants - 12 women with spontaneous abortion (first or second trimester) - 3 women with stillbirth - No differences were found between study groups - 9 babies born with congenital abnormality (3 ginger, 6 B6) (NS) - 6 cases of urogenital disorders - 2 cases of minor gastrointestinal abnormalities -1 case of a minor congenital heart defect	Smith et al., 2004 [79]
To examine the evidence for the safety and effectiveness of ginger for NVP	Women NVP Between 7 and 17 weeks of gestation	*n* = 62 30 placebo 32 ginger	5 biscuits/day (2.5 g of ginger) vs. placebo	-	4 days + follow-up visit 7 days later	Nausea scores: - significantly greater in the ginger group vs. in placebo group (*p* = 0.01) Vomiting episodes: - no significant difference (*p* = 0.243) No vomiting after 4 days: - 34% in ginger group vs. 18% in placebo group Follow-up visits: - 87.5% ginger group reported improvement vs. 70% in placebo group (*p* = 0.043)	In ginger group: - 1 dizziness - 1 heartburn **The side effects reported as minor.** **No abnormal pregnancy and delivery outcome occurred. No infants had any congenital abnormalities recognized.** **All discharged in good condition**	Basirat et al., 2009 [86]
To compare the breast milk volume during the early postpartum period between women receiving dried ginger capsules with those receiving placebo	Women ≥ 37 weeks gestation	*n* = 63 33 placebo 30 ginger	1 g of ginger (500 mg × 2) vs. placebo	Dried ginger powder	7 days	Breast milk volume: - day 3 ginger group has higher milk volume than the placebo group (*p* < 0.01) - day 7, the ginger group does not differ from the placebo group Prolactin levels is similar in both groups	No notable side effects	Paritakul et al., 2016 [74]
To study the efficacy of ginger and dimenhydrinate in the treatment of NVP	Women NVP < 16 weeks of gestation	*n* = 170 85 dimenhydrinate 85 ginger	1 g (500 mg × 2) of ginger vs. 100 mg (50 mg × 2) of dimenhydrinate	-	1 week	Nausea: - the mean score in day 1-7 decreased in both groups - daily mean scores between both groups were not statistically different Vomiting: - frequency of vomiting times in day 1-7 decreased in both groups - daily mean vomiting times in the dimenhydrinate group in day 1-2 were less than the ginger group (*p* < 0.05) - after day 3–7 post treatment, the daily mean vomiting times in both groups were not statistically different	Drowsiness: - 5/85 in the ginger group vs. 66/85 in dimenhydrinate group (*p* < 0.01) Heart burn: - 13/85 in the ginger group vs. 9/85 in dimenhydrinate group (*p* = 0.403) **No other adverse effect was reported in both groups**	Pongrojpaw et al., 2007 [75]
To study the efficacy of ginger and placebo in hyperemesis gravidarum.	Women hyperemesis gravidarum < 20 weeks of gestation	*n* = 27 13 lactose 14 ginger	1 g (250 mg × 4) of ginger vs. placebo	Powdered root	2 × 4 days 2 days washout	The preference: - ginger treatment period was statistically significant (*p* = 0.003) Relief of the hyperemesis symptoms: - significantly greater in ginger group vs. placebo (*p* = 0.035)	One spontaneous abortion, which was not a suspicious high rate of fetal wastage in early pregnancy No side effects were observed All infants were without deformities and discharged in good condition.	Fischer-Rasmussen et al., 1991 [63]
To compare the effectiveness of ginger and acupressure in the treatment of NVP	Women NVP < 16 weeks of gestation	*n* = 143 45 control 48 acupressure	750 mg (250 mg × 3) of ginger vs. acupressure	-	7 days - 3 with no intervention - 4 with treatment	Rhodes index scores: - better in the ginger group vs. acupressure and control (*p* < 0.001) - reduced 49% in ginger group and 29% in acupressure group. - increased up to 0.06% in control group Post hoc test showed significant differences in vomiting, nausea, retching, and total scores between the groups except for vomiting score between acupressure and control groups and for retching score between acupressure and ginger groups	1 case of heartburn with ginger capsules	Saberi et al., 2013 [76]
To determine the effect of ginger to relieve NVP	Women NVP < 16 weeks of gestation	*n* = 106 36 placebo 33 control 37 ginger	750 mg (250 mg × 3) of ginger vs. acupressure	-	7 days - 3 with no intervention - 4 with treatment	Rhodes index scores: - greater in the ginger group vs. placebo and control (*p* < 0.001) - reduced 48% in ginger group,13% in placebo group and -10% in control group Post hoc test showed significant difference between the groups in reduction of vomiting, nausea, retching and total Rhodes Index scores (*p* < 0.001)	1 case of heartburn with ginger capsules	Saberi et al., 2014 [77]
To compare the effects of ginger, pyridoxine (vitamin B6), and placebo for the treatment of NVP	Women between 6 and 16 weeks of pregnancy; mild and moderate NVP	*n* = 77 23 placebo 26 vit. B6 28 ginger	1 g (500 mg × 2) ginger capsules 80 mg (40 mg × 2) Vit. B6 capsules	-	4 days	Rhodes index scores: - ginger > placebo (*p* = 0.039) - Vit. B6 > placebo (*p* = 0.007) - ginger = Vit. B6 (*p* = 0.128) Ginger was more effective for - nausea intensity - nausea distress - distress of vomiting	Ginger is effective and safe	Sharifzadeh et al., 2017 [70]

**Table 10:** Prospective studies investigating the effectiveness and safety of ginger for pregnancy.

Study Type	Objective	Population	Number	Treatment	Ginger Definition	Duration	Results	Adverse Events	Reference
Prospective cohort study (Korean Motherisk Program)	To determine if ginger exposure during pregnancy would increase the risk of adverse fetal and neonatal outcomes	Women Cough and cold preparations (49.7%) Functional gastrointestinal disorders (37.7%) 3 days to 20.7 weeks	159 306 (control group)	Median dose: 470 mg/day Maximum dose: 7.2 g/day	Dried ginger	Median length: 2 days		Spontaneous abortion: - 7 in the ginger group vs. 17 in the control group (NS) Stillbirths: - 2.7% in the ginger group vs. 0.3% in the control group (NS) NICU admission: - 4.7% in the ginger group vs. 1.7% in the control group (NS) The admission rate was marginally different between cases and controls however, no difference was observed when admission rate was compared with that reported in 2012 at the hospital. **They concluded that dried ginger is not a major human teratogen**	Choi et al., 2014 [73]
The Norwegian Mother and Child Cohort study	To evaluate the safety of ginger use during pregnancy on congenital malformations	Women NVP first trimester	*n* = 68 522 - 1020 (use ginger) - 466 (use ginger during 1st trimester)	-	-	-		Not increase the risk of malformations No significant associations between ginger use risk of: - Stillbirth - Perinatal death - Low birth weight - Preterm birth - Low Apgar score	Heitmann et al., 2013 [72]
Prospective cohort study (The Motherisk Program)	The primary objective: - to examine the safety The secondary objective: - to examine the effectiveness of ginger for NVP	Women first trimester	187 (ginger group) 187 (comparison group)	Various types of ginger: - Capsules (49%) - Ginger tea - Fresh ginger - Pickled ginger - Ginger cookies - Ginger candy - Inhaled powdered ginger - Ginger crystals - Sugared ginger	-	Minimum 3 days	Ginger effectiveness scores (overall) on 66 women - 3.6 ± 2.4 (SD) Scale range of effectiveness: - 0 => 29 (43.9%) - 2–4 => 19 (28.8%) - 5–7 => 13 (19.7%) - 8–10 => 6 (7.6%) 0 = No effect; 1–4 = mild effect; 5–7 = moderate effect; 8–10 = best effect.	- 3 spontaneous abortions - 2 stillbirths - 1 therapeutic abortion (Down syndrome) 3 major malformations in the ginger group: - ventricular septal defect - lung abnormality - kidney abnormality (pelviectasis) - 1 idiopathic central precocious puberty at 2 years old No significant differences between the groups in terms of live births, spontaneous abortions, stillbirths, therapeutic abortions, birth weight, or gestational age. Eight sets of twins in the ginger group. More babies < 2.5 Kg in the control group	Portnoi et al., 2003 [71]

**Table 11:** List of adverse effects identified in the different clinical studies.

Adverse Effects Identified (No Significance)	References
Headache, abdominal discomfort, diarrhea, heartburn, spontaneous abortion	Vutyavanich et al., 2001 [80]
Sedation, arrhythmia, heartburn	Chitumma et al., 2007 [81]
Intolerance, allergic reaction, medical assistance, spontaneous abortion	Willets et al., 2003 [85]
Dry retching, vomiting, burning sensation, belching, spontaneous abortion	Smith et al., 2004 [79]
Dizziness, heartburn	Basirat et al., 2009 [86]
Drowsiness, heartburn	Pongrojpaw et al., 2007 [75]
Spontaneous abortion	Ensiyehh et al., 2009 [83]
Spontaneous abortion	Fisher Rasmussen et al., 1991 [63]
Heartburn	Saberie et al., 2013 [76]
Heartburn	Saberie et al., 2014 [77]

## Abbreviations

T&CM	traditional & complementary medicine
CAM	complementary and alternative medicine
USDA	United States Department of Agriculture
INCA	Étude individuelle nationale des consommations alimentaires
ANSES	Agence nationale de sécurité sanitaire de l’alimentation, de l’environnement et du travail (France)
UK	United Kingdom
EU	European Union
USA	United States of America
RCT	randomized clinical trial
EC	European Commission
EFSA	European Food Safety Authority
NVP	nausea and vomiting of pregnancy
BW	Body Weight
ca.	circa
HMPC	Committee on Herbal Medicinal Products
